# The Effects of Receptor Activator of NF-κB Ligand-Binding Peptides on Bone Resorption and Bone Formation

**DOI:** 10.3389/fcell.2021.648084

**Published:** 2021-07-06

**Authors:** Fatma Rashed, Shingo Kamijyo, Yuri Shimizu, Yuna Hirohashi, Masud Khan, Yasutaka Sugamori, Ramachandran Murali, Kazuhiro Aoki

**Affiliations:** ^1^Graduate School of Medical and Dental Sciences, Institute X, Department of Basic Oral Health Engineering, Tokyo Medical and Dental University, Tokyo, Japan; ^2^Department of Oral Biology, Faculty of Dentistry, Damanhour University, El Behera, Egypt; ^3^Department of Dentistry and Oral Surgery, Saitama Medical University, Saitama, Japan; ^4^Biomedical Sciences, Research Division of Immunology, Samuel Oschin Comprehensive Cancer Institute, Cedars-Sinai Medical Center, Los Angeles, CA, United States

**Keywords:** RANKL-binding peptide, bone resorption, bone formation, RANKL-reverse signaling, RANKL clustering, osteoprotegerin

## Abstract

Receptor activator of NF-κB ligand (RANKL)-binding peptides inhibit bone resorption and were recently shown to activate bone formation. The stimulatory mechanism underlying bone formation associated with these peptides was explained as RANKL-reverse signaling, wherein RANKL molecules on osteoblasts work as receptors to stimulate osteoblast differentiation. However, why RANKL-binding peptides stimulate osteoblast differentiation while osteoprotegerin (OPG), which is well known to bind to RANKL, cannot activate osteoblast differentiation has remained unclear. In this mini-review, we introduce three main issues: (1) The inhibitory effects of two RANKL-binding peptides (W9 and OP3-4) on bone resorption; (2) The stimulatory effects of the RANKL-binding peptides on osteoblast differentiation; and (3) The accumulation and membrane clustering of RANKL molecules at the cell surface of osteoblasts as a potential molecular switch stimulating osteoblast differentiation by RANKL-binding peptides.

## Introduction

Several lines of evidence showed that long-term treatment of bisphosphonates, which are bone resorption inhibitors, may cause atypical fracture of the femoral diaphysis due to the building up of micro-cracks induced by the suppression of bone turnover and accumulation of the old bone (Saita et al., 2015). Anti-RANKL antibody is also known to reduce bone formation via the coupling of resorption and formation during bone remodeling (Furuya et al., 2011; Reginster, 2011). While these anti-resorptive drugs have been shown to improve bone mineral density and reduce fracture risks in osteoporotic patients, adding bone formation ability to bone resorption inhibitors may be a useful feature for a novel drug for treating osteoporosis (Lewiecki, 2011). Recently, two anabolic therapies were developed: Intermittent parathyroid hormone (teriparatide) and anti-sclerostin antibody (romosozumab). However, one of their shortcomings is that their treatment period is limited, a maximum of 2 years for parathyroid hormone and 1 year for the anti-sclerostin antibody. In some cases, teriparatide treatment is shown to increase cortical porosity depending on the time frame and frequency of the treatment. Furthermore, antibody therapy is considered to be a reversible treatment, with short efficacy after therapy discontinuation (Appelman-Dijkstra and Papapoulos, 2018; Lewiecki et al., 2019). Therefore, an alternative bone anabolic reagent with a novel signaling pathway to stimulate bone formation is needed.

We have previously shown that the RANKL-binding peptides WP9QY (W9) and OP3-4 can suppress bone resorption (Cheng et al., 2004; Aoki et al., 2006). For instance, W9 inhibited RANKL-induced osteoclast formation *in vitro* and prevented bone resorption *in vivo* induced by ovariectomy and low dietary calcium. Likewise, OP3-4, a cyclic peptide that was originally designed from the RANKL-binding site on OPG as a template (Table 1A), inhibited RANKL-induced osteoclastogenesis and prevented bone resorption in a murine ovariectomized model (Table 1B). However, surprisingly, W9 and OP3-4 were also found to exert stimulatory effects on bone formation in addition to inhibiting bone resorption (Kato et al., 2015; Furuya et al., 2013). Herein, we report the effect of these RANKL-binding peptides on bone resorption and bone formation, mainly focusing on the peptides’ stimulatory mechanism on bone formation compared with osteoprotegerin (OPG), which cling to RANKL (Simonet et al., 1997; Sone et al., 2019).

**TABLE 1:** Evidence supporting the effects of the receptor activator of NF-κB ligand (RANKL)-binding peptides W9 and OP3-4.

	**Subject**	**Related figures**	**References**
**A**	***OP3-4 structure:*** OP3-4 was designed based on the RANKL-binding site on OPG as a template. -*Molecular weight:*1448 (**11** amino acid) *YCEIEFCYLIR*	**Figure 1**	Cheng et al., 2004
**B**	***OP3-4 blocked ovariectomy-induced bone loss.***	**Figure 5**	Cheng et al., 2004
**C**	***Alignment of TNF receptor superfamily and WP9QY (W9) peptide:*** W9 is a molecule designed using the TNF-α binding site on the TNF type 1 receptor as a template. It binds to both TNF-α and RANKL. -*Molecular weight:*1226 (**9** amino acids) *YCWSQYLCY*	**Figure 1A**	Aoki et al., 2006
**D**	***W9 blocks RANKL-induced signaling:*** In the osteoclast precursor RAW 264.7 cell line, W9 inhibited the soluble RANKL-induced translocation of p65, a significant component of NF-κB, to the nucleus.	**Figure 3A**	Aoki et al., 2006
***E***	***W9 cannot separateRANK and RANKL***: A competition assay measuring the binding of RANKL to the soluble RANK-coated surface with W9, compared with an assay using OPG.	**Figures 2B,C**	Aoki et al., 2006
***F***	***Conformational changes of RANK-RANKL complex by W9:*** Molecular modeling revealed the conformational changes in the RANK-RANKL complex induced by W9.	**Figure 1C**	Aoki et al., 2006
***G***	***OP3-4 activated bone formation in the collagen-induced arthritis model:*** The RANKL-binding peptide prevents a reduction in bone formation in the arthritis model.	**Figures 6B,F**	Kato et al., 2015
***H***	***OP3-4 and W9 had similar stimulatory effects on osteoblast differentiation in vitro and on BMP-2-induced bone formation in vivo.***	**Figures 1–3**	Sugamori et al., 2016
***I***	***OP3-4 and W9 enhanced the upstream and downstream signaling of mTORC1 activity in ST2 cells.***	**Figure 5**	Sugamori et al., 2016
***J***	***The stimulatory effects of RANKL-binding peptide W9 on osteoblast differentiation were blunted in RANKL-deficient osteoblasts:*** The RANKL-binding peptide enhanced osteoblast differentiation in a RANKL-dependent manner.	**Figure 3A** Extended data	Ikebuchi et al., 2018
***K***	***The RANKL-binding peptide OP3-4 enhanced ALP activity, but the RANKL-binding molecule OPG did not use ST2 cells.***	**Figure 1D**	Sone et al., 2019
***L***	***The RANKL-binding peptide OP3-4 increased the amount of membrane- RANKL, while OPG did not:*** OP3-4 increased the membrane fraction of RANKL both 6 and 24 h after stimulation of the peptide, but OPG did not.	**Figure 2**	Sone et al., 2019
***M***	***OPG clung to RANKL and disturbed clustering while RANKL-binding peptides did not:*** HS-AFM imaging revealed the formation of clusters of RANKL molecules and highly flexible OPG clinging to RANKL.	**Figure 3** Video S1A_V2 Video S1B_V2	Sone et al., 2019
***N***	***Artificial induction of molecular clustering of RANKL may lead to the enhancement of osteoblast differentiation, even when OPG is used:*** Inducing pentameric assembly of OPG-Fc-RANKL complex by IgM could lead to osteoblast differentiation and enhancement of RANKL accumulation in ST2 cells.	**Figure 4**	Sone et al., 2019
***O***	***W9 exerted similar or even stronger inhibitory effects on bone destruction than anti-TNF antibody***	**Figure 5**	Saito et al., 2007

### The RANKL-Binding Peptide W9 Inhibits RANK-Downstream Signaling by Inducing Topological Changes in the RANK Structure

W9 is a cyclic peptide composed of nine amino acids, originally designed as a tumor necrosis factor (TNF)-α antagonist (Takasaki et al., 1997). We aligned the amino acid sequence in the cysteine-rich domain of different TNF-receptor-superfamily members, such as TNF type 1 receptor, RANK, and OPG; we found that five amino acids, used to design W9 peptide on TNF type 1 receptor, were homologous with corresponding residues in RANK, but not in OPG (Table 1C). Additionally, the soluble RANKL (sRANKL)-induced nuclear translocation of p65, a major component of NF-κB, was blocked by W9 (Table 1D). Using surface plasmon resonance (SPR) assay, the peptide-sequence specificity that features RANKL-binding affinity was confirmed. Hereby, changing the tyrosine residue in position 6 (Tyr6) to asparagine (Y6N) of W9 reduced the binding to RANKL by more than 70% at concentrations up to 100μM.

**FIGURE 1:**
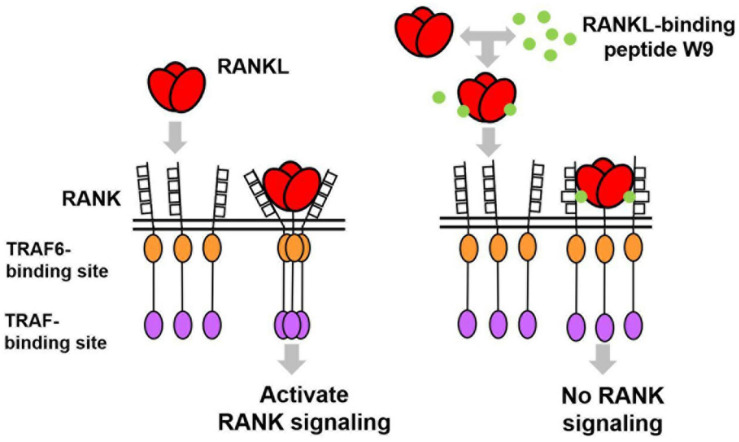
A diagram illustrating the proposed mechanism of RANK downstream signaling inhibition, which is induced by the receptor activator of NF-κB ligand (RANKL)-binding peptide. The peptide-induced conformational changes of RANK, especially the region close to the lipid bilayer membrane (shown with double lines), could block the downstream signaling of RANK, thereby allowing RANKL to maintain contact with RANK.

In general, an antagonist interferes with receptor–ligand interaction separating a ligand and its receptor. OPG is considered an antagonist since it was shown to separate RANK and RANKL and, consequently, prevent trimer formation of RANK, leading to the inactivation of receptor RANK downstream signaling (Lacey et al., 2012). However, a competition assay using SPR showed that W9 was not a typical antagonist since it could not separate receptor RANK and ligand RANKL (Table 1E). This left us wondering how this peptide could block the downstream signaling of RANK while maintaining the binding of RANKL with RANK. The molecular modeling revealed that W9 induced conformational changes in the RANK–RANKL complex, especially the trimer formation of RANK (Table 1F). These results suggested that the peptide could block the signaling downstream of the receptor RANK by inducing conformational changes of RANK extracellular domain (Table 1F). Based on that molecular modeling, our proposed mechanism for W9 to block RANK downstream signaling is shown in Figure 1.

### RANKL-Binding Peptides Stimulate Bone Formation in a RANKL-Dependent Manner

In Furuya et al. (2013) first demonstrated the stimulatory effects of W9 on bone formation and osteoblast differentiation, both *in vitro* and *in vivo*, and obtained by subcutaneous injections three times daily for 5 days. OP3-4 treatment did not reduce the inflammatory indices in a murine arthritis model and not only prevented periarticular bone loss at the tibial metaphysis but promoted the bone formation parameters in the secondary spongiosa at the tibial metaphysis in collagen-induced arthritic (CIA) mice (Table 1G). The peptide was delivered using subcutaneously implanted osmotic minipumps at a concentration of 9 or 18 mg/kg/day. Furthermore, OP3-4 and W9 had stimulatory effects on osteoblast differentiation *in vitro* and BMP-2-induced bone formation *in vivo* (Table 1H). However, the precise mechanism underlying the anabolic effects induced by the RANKL-binding peptides was not clarified by these studies.

Ikebuchi et al. (2018) used vesicles containing RANK secreted from osteoclasts to induce RANKL reverse signaling in osteoblasts. The signaling from an Src family kinase to mammalian target of rapamycin complex 1 (mTORC1) signaling (Saksela et al., 1995; Cuevas et al., 2001) and the signaling from mTORC1 to the activation of alkaline phosphatase activity through runt-related transcription factor 2 (Runx2) (Singha et al., 2008) had already been reported. Ikebuchi et al. (2018) showed the importance of the proline-rich motif in the intracellular domain of the membrane-bound RANKL on osteoblasts, which is a signaling motif, leading to the activation of mTORC1 activity and alkaline phosphatase activity. A mouse model with a one-point mutation of proline to alanine (Pro29Ala) in the RANKL intracellular domain was used to clarify the RANKL-reverse signaling, wherein RANKL works as a receptor on the osteoblast surface. Binding to the membrane-bound RANKL directly activated the intracellular signaling of RANKL in osteoblasts.

**FIGURE 2:**
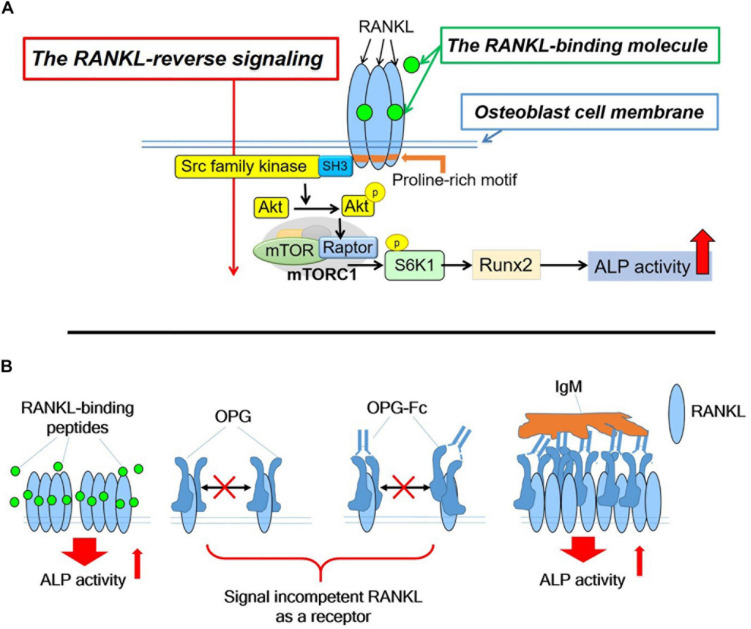
**(A)**. Hypothetical diagram showing the stimulatory mechanism of the OP3-4 and W9 on osteoblast differentiation through RANKL-reverse signaling. SFKs, Src family kinases; mTORC1, the mammalian target of rapamycin complex 1; S6K1, ribosomal protein S6 kinase 1. Although the intracellular domain of RANKL is short, proline-rich motif of the domain is shown to be important to activate the RANKL-reverse signaling, which leads to stimulate early osteoblast differentiation. The proline-rich motif activates a Src-family kinase by associating with SH3 domain followed by Akt and mTORC1 signaling, leading to the activation of runt-related transcription factor 2 (Runx2) and alkaline phosphatase (ALP) activity **(B)**. The RANKL-accumulation and continued clustering of RANKL molecules on the cell- membrane of osteoblasts could be a signaling switch to stimulate the ALP activity. The RANKL molecule itself has the characteristic features of clustering (Table 1M). After the peptides bind to membrane-bound RANKL, the accumulation of RANKL molecules takes place without disturbing the characteristic features of the RANKL molecules, thus resulting in the enhancement of the alkaline phosphatase activity leading to early osteoblast differentiation. OPG alone and OPG-Fc alone do not induce RANKL accumulation (Table 1L) since they prevent RANKL-clustering (Table 1M). However, when IgM induced the pentameric assembly of OPG-Fc, then RANKL accumulation took place. Eventually, both alkaline phosphatase and other early osteoblast differentiation markers were observed to increase (Table 1N).

The RANKL-binding peptides (OP3-4 and W9) had previously been shown also to activate Akt phosphorylation and S6k1 phosphorylation (upstream and downstream signaling of mTORC1 activity, respectively) (Table 1I). Furthermore, the stimulatory effects of RANKL-binding peptide on osteoblast differentiation were blunted in RANKL-deficient osteoblasts (Table 1J). These results suggest that the RANKL-binding peptides stimulate osteoblast differentiation in a RANKL-dependent manner, which means that downstream signaling and alkaline phosphatase activity (ALP) activity cannot be activated in the absence of RANKL. Figure 2A shows the proposed mechanism underlying the stimulation of osteoblast differentiation by W9 and OP3-4, based on our findings that the proline-rich motif in the RANKL-intracellular domain is important for inducing the RANKL-reverse signaling and stimulating early osteoblast differentiation upon stimulation of RANKL. RANKL (Pro29Ala) mutant mice have an osteopenic phenotype and reduced bone formation activity compared with littermate control mice (Ikebuchi et al., 2018). Generally, bone formation is enhanced after the stimulation of bone resorption due to the coupling mechanism from bone resorption and bone formation; however, bone formation in the mutant mice was not enhanced when bone resorption was activated in the sRANKL-induced osteopenic model. Ikebuchi et al. (2018) further showed that signaling downstream of Src family kinase was reduced in the RANKL mutant mice. The RANKL-binding molecule-induced phosphorylation of Akt and S6k1 was reduced in the osteoblasts derived from the mutant mice compared with the littermate controls. Therefore, the proline-rich motif in the RANKL cytoplasmic tail was proven to be essential for activating the intracellular signal transduction pathway, corresponding to RANKL-reverse signaling (Ikebuchi et al., 2018).

### The Conditions Where the RANKL-Binding Molecules Activate the RANKL-Reverse Signaling

OP3-4 and W9 enhanced alkaline phosphatase activity, but OPG, which also binds to RANKL, did not, indicating that not all RANKL-binding molecules stimulate osteoblast differentiation (Table 1K). The high turnover rate of bone remodeling in OPG-deficient mice compared with wild-type mice supports the lack of anabolic effects of OPG on bone formation (Fei et al., 2010). Therefore, we were left wondering about the differences in the effects between RANKL-binding peptides and OPG on RANKL-reverse-signaling activation to stimulate osteoblast differentiation.

We assumed that one condition that can activate the RANKL-reverse signaling is RANKL accumulation at the osteoblasts’ plasma membrane, while another condition is RANKL clustering. First, the accumulation of RANKL molecules is thought to be necessary to induce RANKL molecule clustering since clustering is unlikely to proceed unless the amount of RANKL on the osteoblast membrane increases. RANKL molecule was shown to translocate from the Golgi apparatus to the plasma membrane and accumulate on the cell surface of ST2 cells, osteoblastic cell line, upon the stimulation of RANK-coated beads (Kariya et al., 2009). In the same way, we found that RANKL molecule accumulated on the plasma membrane on ST2 cells upon the stimulation of RANKL-binding peptide, either OP3-4 or W9 (Table 1L). Since OPG did not increase the amount of membrane- RANKL on osteoblasts (Table 1L), these results could be evidence to explain the difference between the RANKL-binding peptides and OPG. Second, the clustering of RANKL molecules could be another evidence as described next. It has been shown that the clustering of membrane-bound receptors leads to cell activation (Boger and Goldberg, 2001; Lacey et al., 2012). The distance between the receptors was also found to be crucial for activating the erythropoiesis signal (Erickson-Miller et al., 2005). Furthermore, Kosmides et al. found that inducing artificial clusters of co-stimulatory molecules on the immune cell membrane by magnetic force enhanced T-cells’ proliferative activity (Kosmides et al., 2018). Therefore, we hypothesized that clustering of membrane-bound RANKL molecules on osteoblasts would induce a switch to activate osteoblast differentiation in addition to the accumulation of membrane-RANKL.

### OPG Clung to RANKL and Disturbed Clustering While RANKL-Binding Peptides Did Not

Next, we investigated the difference in the molecular features between RANKL molecules with the RANKL-binding peptide and those with OPG in a cell-free system. The RANKL molecules and OPG were observed on mica using a high-speed atomic force microscope (HS-AFM), revealing the characteristic features at the protein’s single-molecule level. HS-AFM is a powerful instrument that enables the direct imaging of protein molecules in their dynamic action without disturbing the molecules’ function. HS-AFM revealed that the highly flexible OPG clung to the RANKL molecule and disturbed clustering, while the RANKL-binding peptide OP3-4 did not. Furthermore, the molecular features of RANKL itself showed easy clustering, which did not change with the addition of OP3-4. However, OPG appears to move around RANKL while binding to it, suggesting that OPG inhibits the clustering formation of RANKL molecules (Table 1M).

### Artificial Induction of Molecular Clustering of RANKL Could Lead to the Enhancement of Osteoblast Differentiation, Even When Osteoprotegerin Was Used

As we mentioned earlier, RANKL-binding peptides increase ALP activity in the cell line and further increase membrane RANKL levels, but OPG does not. Further verification in a cell-free system revealed that the RANKL molecule itself has the property of easily becoming clustered, suggesting that RANKL-binding peptides do not inhibit clustering while OPG does. Therefore, the key to inducing osteoblast differentiation seems to lie in the clustering of RANKL. Artificial cluster induction using IgM to promote pentameric assembly of RANKL–OPG-Fc complex enhances early osteoblast differentiation (Table 1N). These results suggest that clustering formation may be a pivotal switch for enhancing osteoblast differentiation by RANKL-binding molecules (Figure 2B). Simultaneously, the amount of RANKL on the osteoblast membrane also increased after the induction of pentameric assembly of the RANKL–OPG-Fc complex by IgM (Table 1N).

## Discussion

The cyclic peptide W9 was designed to mimic the most critical TNF-α recognition loop on the TNF type 1 receptor, and it prevents interactions of TNF-α with its receptor. Even though the W9 peptide had a weaker inhibitory effect on inflammation than the anti-TNF antibody, W9 exerted similar or even stronger inhibitory effects on bone destruction than the anti-TNF antibody (Table 1O). Similarly, the cyclic peptide OP3-4, designed to mimic the binding site of the RANKL molecule on OPG, was shown to cling to RANKL, thereby inhibiting osteoclastogenesis. Kato et al. (2015). further clarified using dynamic histomorphometry that OP3-4 decreased the inhibition of bone formation caused by CIA (Table 1G).

While the RANKL-binding peptides have been found to stimulate bone formation, neither OPG nor anti-RANKL antibodies were able to enhance bone formation (Dore et al., 2010). However, a recent paper claimed that anti-RANKL-antibody might also stimulate bone formation using RANKL-reverse signaling (Wang et al., 2020). As anti-RANKL antibody was found to decrease levels of bone formation markers in clinical studies (Cummings et al., 2009; Ominsky et al., 2015), further studies are necessary to clarify whether or not anti-RANKL antibody can stimulate bone formation in addition to inhibiting bone resorption. Furthermore, the ideal timing of inducing the accumulation of RANKL molecules and RANKL clustering to activate RANKL-reverse signaling should be investigated.

The pivotal role of RANKL expressed on osteocytes in osteoclast formation was clarified using a cell-specific RANKL-deficient mouse (Nakashima et al., 2011; Xiong et al., 2011), while osteoblasts’ RANKL was shown to be involved in a role other than osteoclastogenesis. Ikebuchi et al. (2018) revealed the physiological role of RANKL on osteoblasts, which works as a receptor to stimulate bone formation using RANKL-reverse signaling (Figure 2A). The small extracellular vesicles containing RANK secreted from mature osteoclasts (OC-SEVs) were shown to work as a ligand of the acceptor RANKL on osteoblasts. As we described earlier, a coupling phenomenon from bone resorption to bone formation was abolished when RANKL point-mutation mice (Pro29Ala) were used in the RANKL-induced osteopenic model. These results suggest that RANKL on osteoblasts works as a receptor to stimulate bone formation even under pathophysiological conditions. Therefore, RANKL on osteoblasts could be a potential pharmacological target to recover the bone remodeling balance between bone resorption and bone formation. The RANKL-binding molecule could be an effective reagent for the treatment of bone diseases such as postmenopausal osteoporosis. We also expect a RANKL-binding molecule to stimulate local bone formation at the fracture site or the site necessary for bone regeneration. Therefore, RANKL-reverse signaling can be a ground-breaking stone for developing anabolic bone reagent.

The peptides potentiate the effect by bone morphogenetic protein (BMP)-2 as subcutaneous injections of W9 promoted bone mineral density, increased the mineral apposition rate of the femur in mice and accelerated BMP-2-induced ectopic bone formation (Furuya et al., 2013). Of note, W9 promoted BMP-2-induced bone formation by a mechanism other than the antagonism of TNF-α action, as synergistic effects still existed when TNF type 1 receptor-deficient mice or TNF-α-deficient mice were used to establish a BMP-2-induced subcutaneous bone formation model (Khan et al., 2013). In the same manner, we demonstrated the synergistic effects of RANKL-binding peptides with BMP-2 using a sub-periosteally injected bone formation model of the murine maxilla (Uehara et al., 2016; Keo et al., 2020), a tooth extraction model at the incisor socket (Arai et al., 2016), and a model of standard ectopic bone formation at a calvarial bone defect (Khan et al., 2013; Sugamori et al., 2016). Although W9 peptide stimulation has been shown to enhance the phosphorylation of Smad 1 and 5 (Furuya et al., 2013), further studies are necessary to clarify the cross-talk between BMP-2 signaling and RANKL-reverse signaling.

## Conclusion

Induction of accumulation of RANKL molecules and the RANKL clustering at the osteoblast membrane might be a new therapeutic strategy for developing bone anabolic reagents.

## Author Contributions

YH and YS drafted the present manuscript. MK and SK contributed to the conceptual idea, performed database search, analyzed data, and wrote the manuscript. FR and KA received a grant for this project, conceived the idea, reviewed the drafts, and supervised this manuscript’s writing process and editing. YS and RM gave suggestions and significantly revised and refined the manuscript. All authors read and approved the final manuscript.

## Conflict of Interest

The authors declare that the research was conducted in the absence of any commercial or financial relationships that could be construed as a potential conflict of interest.

## References

[B1] Aoki K. Saito H. Itzstein C. Ishiguro M. Shibata T. Blanque R. (2006). A TNF receptor loop peptide mimic blocks RANK ligand-induced signaling, bone resorption, and bone loss. *Journal of Clinical Investigation* 116 1525–1534. 10.1172/JCI22513 16680194 PMC1448165

[B2] Appelman-Dijkstra N. M. Papapoulos S. E. (2018). Clinical advantages and disadvantages of anabolic bone therapies targeting the WNT pathway. *Nature Reviews Endocrinology* 14 605–623. 10.1038/s41574-018-0087-0 30181608

[B3] Arai Y. Aoki K. Shimizu Y. Tabata Y. Ono T. Murali R. (2016). Peptide-induced de novo bone formation after tooth extraction prevents alveolar bone loss in a murine tooth extraction model. *European Journal of Pharmacology* 782 89–97. 10.1016/j.ejphar.2016.04.049 27118173

[B4] Boger D. L. Goldberg J. (2001). Cytokine receptor dimerization and activation: Prospects for small molecule agonists. *Bioorganic and Medicinal Chemistry* 9 557–562. 10.1016/S0968-0896(00)00276-5 11310589

[B5] Cheng X. Kinosaki M. Takami M. Choi Y. Zhang H. Murali R. (2004). Disabling of Receptor Activator of Nuclear Factor-κB (RANK) Receptor Complex by Novel Osteoprotegerin-like Peptidomimetics Restores Bone Loss in Vivo. *Journal of Biological Chemistry* 279 8269–8277. 10.1074/jbc.M309690200 14679212

[B6] Cuevas B. D. Lu Y. Mao M. Zhang J. LaPushin R. Siminovitch K. (2001). Tyrosine Phosphorylation of p85 Relieves Its Inhibitory Activity on Phosphatidylinositol 3-Kinase. *Journal of Biological Chemistry* 276 27455–27461. 10.1074/jbc.M100556200 11337495

[B7] Cummings S. R. Martin J. S. McClung M. R. Siris E. S. Eastell R. Reid I. R. (2009). Denosumab for prevention of fractures in postmenopausal women with osteoporosis. *Obstetrical and Gynecological Survey* 64 805–807. 10.1097/01.ogx.0000363236.41902.96 19671655

[B8] Dore R. K. Cohen S. B. Lane N. E. Palmer W. Shergy W. Zhou L. (2010). Effects of denosumab on bone mineral density and bone turnover in patients with rheumatoid arthritis receiving concurrent glucocorticoids or bisphosphonates. *Ann Rheum Dis.* 69 872–875. 19734132 10.1136/ard.2009.112920 PMC6909937

[B9] Erickson-Miller C. L. DeLorme E. Tian S. S. Hopson C. B. Stark K. Giampa L. (2005). Discovery and characterization of a selective, nonpeptidyl thrombopoietin receptor agonist. *Experimental Hematology* 33 85–93. 10.1016/j.exphem.2004.09.006 15661401

[B10] Fei Q. Guo C. Xu X. Gao J. Zhang J. Chen T. (2010). Osteogenic growth peptide enhances the proliferation of bone marrow mesenchymal stem cells from osteoprotegerin-deficient mice by CDK2/cyclin A. *Acta Biochimica et Biophysica Sinica* 42 801–806. 10.1093/abbs/gmq086 20926513

[B11] Furuya Y. Inagaki A. Khan M. Mori K. Penninger J. M. Nakamura M. (2013). Stimulation of bone formation in cortical bone of mice treated with a receptor activator of nuclear Factor-B Ligand (RANKL)-binding peptide that possesses osteoclastogenesis inhibitory activity. *Journal of Biological Chemistry* 288 5562–5571. 10.1074/jbc.M112.426080 23319583 PMC3581422

[B12] Furuya Y. Mori K. Ninomiya T. Tomimori Y. Tanaka S. Takahashi N. (2011). Increased bone mass in mice after single injection of anti-receptor activator of nuclear factor-κB ligand-neutralizing antibody: Evidence for bone anabolic effect of parathyroid hormone in mice with few osteoclasts. *Journal of Biological Chemistry* 286 37023–37031. 10.1074/jbc.M111.246280 21862583 PMC3196100

[B13] Ikebuchi Y. Aoki S. Honma M. Hayashi M. Sugamori Y. Khan M. (2018). Coupling of bone resorption and formation by RANKL reverse signalling. *Nature* 561 195–200. 10.1038/s41586-018-0482-7 30185903

[B14] Kariya Y. Honma M. Aoki S. Chiba A. Suzuki H. (2009). Vps33a mediates RANKL storage in secretory lysosomes in osteoblastic cells. *Journal of Bone and Mineral Research* 24 1741–1752. 10.1359/jbmr.090409 19419298

[B15] Kato G. Shimizu Y. Arai Y. Suzuki N. Sugamori Y. Maeda M. (2015). The inhibitory effects of a RANKL-binding peptide on articular and periarticular bone loss in a murine model of collagen-induced arthritis: A bone histomorphometric study. *Arthritis Research and Therapy* 17 753–758. 10.1186/s13075-015-0753-8 26373710 PMC4570694

[B16] Keo P. Matsumoto Y. Shimizu Y. Nagahiro S. Ikeda M. Aoki K. (2020). A pilot study to investigate the histomorphometric changes of murine maxillary bone around the site of mini-screw insertion in regenerated bone induced by anabolic reagents. *European Journal of Orthodontics* 2020 018. 10.1093/ejo/cjaa018 32202621

[B17] Khan A. A. M. Alles N. Soysa N. S. Mamun A. Al, Nagano K. (2013). The local administration of TNF-α and RANKL antagonist peptide promotes BMP-2-induced bone formation. *Journal of Oral Biosciences* 55 47–54. 10.1016/j.job.2012.12.005

[B18] Kosmides A. K. Necochea K. Hickey J. W. Schneck J. P. (2018). Separating T Cell Targeting Components onto Magnetically Clustered Nanoparticles Boosts Activation. *Nano Letters* 18 1916–1924. 10.1021/acs.nanolett.7b05284 29488768 PMC6707078

[B19] Lacey D. L. Boyle W. J. Simonet W. S. Kostenuik P. J. Dougall W. C. Sullivan J. K. (2012). Bench to bedside: Elucidation of the OPG-RANK-RANKL pathway and the development of denosumab. *Nature Reviews Drug Discovery* 11 401–419. 10.1038/nrd3705 22543469

[B20] Lewiecki E. M. (2011). New targets for intervention in the treatment of postmenopausal osteoporosis. *Nature Reviews Rheumatology* 7 631–638. 10.1038/nrrheum.2011.130 21931340

[B21] Lewiecki E. M. Dinavahi R. V. Lazaretti-Castro M. Ebeling P. R. Adachi J. D. Miyauchi A. (2019). One Year of Romosozumab Followed by Two Years of Denosumab Maintains Fracture Risk Reductions: Results of the FRAME Extension Study. *Journal of Bone and Mineral Research* 34 419–428. 10.1002/jbmr.3622 30508316

[B22] Nakashima T. Hayashi M. Fukunaga T. Kurata K. Oh-Hora M. Feng J. Q. (2011). Evidence for osteocyte regulation of bone homeostasis through RANKL expression. *Nature Medicine* 17 1231–1234. 10.1038/nm.2452 21909105

[B23] Ominsky M. S. Brown D. L. Van G. Cordover D. Pacheco E. Frazier E. (2015). Differential temporal effects of sclerostin antibody and parathyroid hormone on cancellous and cortical bone and quantitative differences in effects on the osteoblast lineage in young intact rats. *Bone* 81 380–391. 10.1016/j.bone.2015.08.007, 26261096

[B24] Reginster J.-Y. (2011). Antifracture Efficacy of Currently Available Therapies for Postmenopausal Osteoporosis. *Drugs* 71 65–78. 10.2165/11536850-000000000-00000 21175240

[B25] Saita Y. Kaneko K. Ishijima M. (2015). Atypical femoral fractures and bisphosphonate use: Current evidence and clinical implications. *Therapeutic Advances in Chronic Disease* 6 185–193. 10.1177/2040622315584114 26137208 PMC4480549

[B26] Saito H. Kojima T. Takahashi M. Horne W. C. Baron R. Amagasa T. (2007). A tumor necrosis factor receptor loop peptide mimic inhibits bone destruction to the same extent as anti-tumor necrosis factor monoclonal antibody in murine collagen-induced arthritis. *Arthritis Rheum.* 56, 1164–1174. 10.1002/art.22495 17393436

[B27] Saksela K. Cheng G. Baltimore D. (1995). Proline-rich (PxxP) motifs in HIV-1 Nef bind to SH3 domains of a subset of Src kinases and are required for the enhanced growth of Nef+ viruses but not for down-regulation of CD4. *EMBO Journal* 14 484–491. 10.1002/j.1460-2075.1995.tb07024.x PMC398106 7859737

[B28] Simonet W. S. Lacey D. L. Dunstan C. R. Kelley M. Chang M. S. Lüthy R. (1997). Osteoprotegerin: A novel secreted protein involved in the regulation of bone density. *Cell* 89 309–319. 10.1016/S0092-8674(00)80209-3 9108485

[B29] Singha U. K. Jiang Y. Yu S. Luo M. Lu Y. Zhang J. (2008). Rapamycin inhibits osteoblast proliferation and differentiation in MC3T3-E1 cells and primary mouse bone marrow stromal cells. *Journal of Cellular Biochemistry* 103 434–446. 10.1002/jcb.21411 17516572

[B30] Sone E. Noshiro D. Ikebuchi Y. Nakagawa M. Khan M. Tamura Y. (2019). The induction of RANKL molecule clustering could stimulate early osteoblast differentiation. *Biochemical and Biophysical Research Communications* 509 435–440. 10.1016/j.bbrc.2018.12.093 30594398

[B31] Sugamori Y. Mise-Omata S. Maeda C. Aoki S. Tabata Y. Murali R. (2016). Peptide drugs accelerate BMP-2-induced calvarial bone regeneration and stimulate osteoblast differentiation through mTORC1 signaling. *BioEssays* 38 717–725. 10.1002/bies.201600104 27345003 PMC5094554

[B32] Takasaki W. Kajino Y. Kajino K. Murali R. G. M. (1997). Structure–based design and characterization of exocyclic peptidomimetics that inhibit TNFα binding to its receptor. *Nature Biotechnology* 15 1266–1270. 10.1038/nbt1197-1266 9359109

[B33] Uehara T. Matsui M. Tabata Y. Murali R. Miyashin M. Aoki K. (2016). Delivery of RANKL-Binding Peptide OP3-4 Promotes BMP-2–Induced Maxillary Bone Regeneration. *Journal of Dental Research* 95 665–672. 10.1177/0022034516633170 27006466

[B34] Wang L. Huang B. Chen X. Su J. (2020). New insight into unexpected bone formation by denosumab. *Drug Discovery Today* 25 1919–1922. 10.1016/j.drudis.2020.09.001 32916270

[B35] Xiong J. Onal M. Jilka R. L. Weinstein R. S. Manolagas S. C. Brien C. A. O. (2011). Matrix-embedded cells control osteoclast formation. *Nature Medicine* 17 1235–1242. 10.1038/nm.2448 21909103 PMC3192296

